# Supplementary figures and images for: DHODH inhibition enhances the efficacy of immune checkpoint blockade by increasing cancer cell antigen presentation
Source: eLife. 2024 Jul 8;12:RP87292. doi: 10.7554/eLife.87292 (PMC11230627; doi:10.7554/eLife.87292)

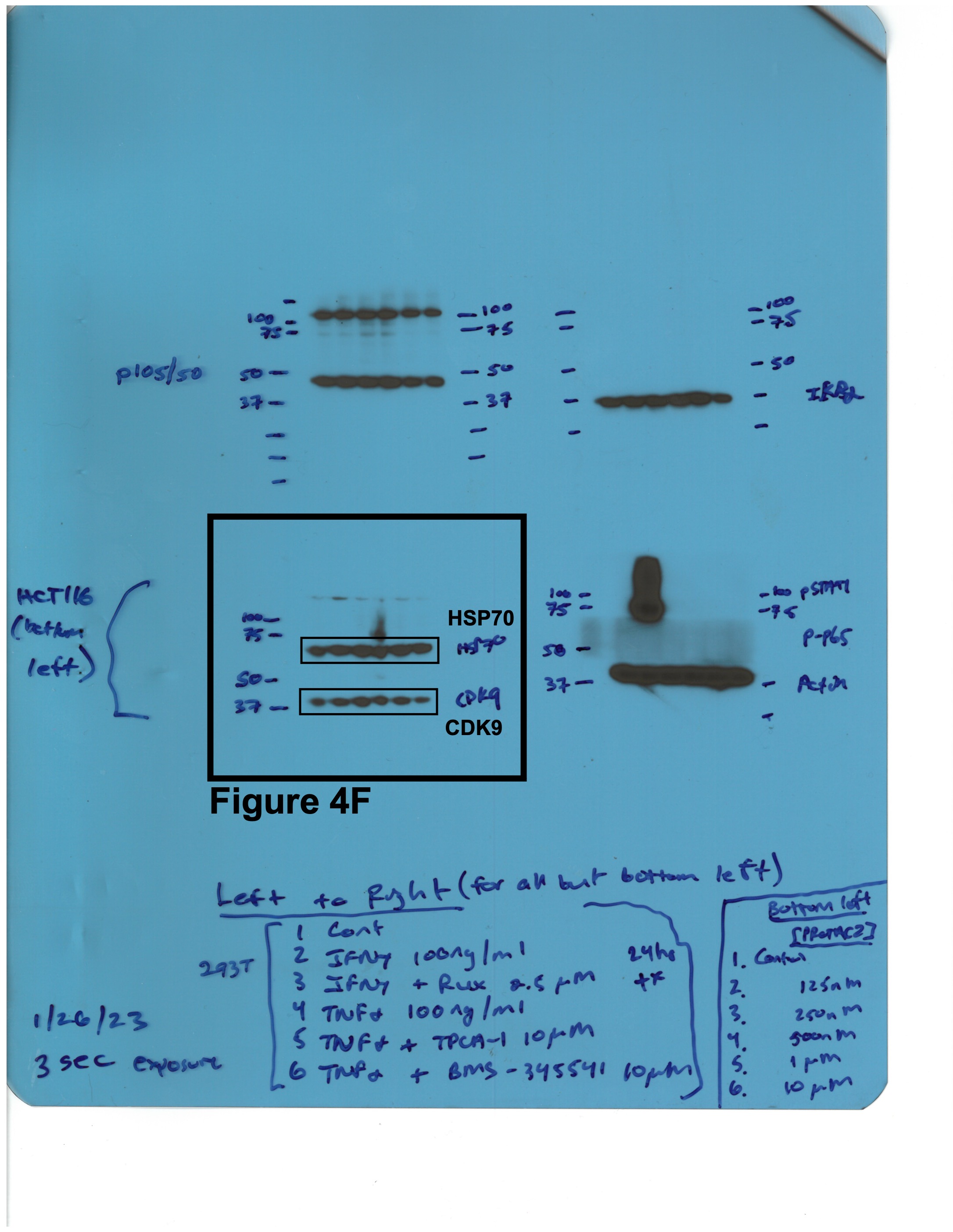

Supplement: Figure 4—source data 2. [file elife-87292-fig4-data2.zip › Figure 4 - source data 2/Figure 4F uncropped with bands highlighted.jpg]
